# Risk factors for severe acute lower respiratory infections in children – a systematic review and meta-analysis

**DOI:** 10.3325/cmj.2013.54.110

**Published:** 2013-04

**Authors:** Stewart Jackson, Kyle H. Mathews, Dražen Pulanić, Rachel Falconer, Igor Rudan, Harry Campbell, Harish Nair

**Affiliations:** 1 Centre for Population Health Sciences, University of Edinburgh, Medical School, Edinburgh, Scotland, UK; 2 Clinical Hospital Centre Zagreb, Zagreb, Croatia, and Faculty of Medicine Osijek, J.J. Strossmayer University of Osijek, Osijek, Croatia; 3 Public Health Foundation of India, New Delhi, India

## Abstract

**Aim:**

To identify the risk factors in children under five years of age for severe acute lower respiratory infections (ALRI), which are the leading cause of child mortality.

**Methods:**

We performed a systematic review of published literature available in the public domain. We conducted a quality assessment of all eligible studies according to GRADE criteria and performed a meta-analysis to report the odds ratios for all risk factors identified in these studies.

**Results:**

We identified 36 studies that investigated 19 risk factors for severe ALRI. Of these, 7 risk factors were significantly associated with severe ALRI in a consistent manner across studies, with the following meta-analysis estimates of odds ratios (with 95% confidence intervals): low birth weight 3.18 (1.02-9.90), lack of exclusive breastfeeding 2.34 (1.42-3.88), crowding – more than 7 persons per household 1.96 (1.53-2.52), exposure to indoor air pollution 1.57 (1.06-2.31), incomplete immunization 1.83 (1.32-2.52), undernutrition – weight-for-age less than 2 standard deviations 4.47 (2.10-9.49), and HIV infection 4.15 (2.57-9.74).

**Conclusion:**

This study highlights the role of the above seven risk factors in the development of severe pneumonia in under-five children. In addition, it emphasizes the need for further studies investigating other potential risk factors. Since these risk factors are potentially preventable, health policies targeted at reducing their prevalence provide a basis for decreasing the burden of childhood pneumonia.

Pneumonia is one of the leading causes of morbidity and mortality globally in young children aged below five years (1). It is estimated that approximately 156 million cases of pneumonia occur annually in young children, resulting in approximately 1.4 million deaths (2,3). Over the past two decades, there were several attempts to investigate an association between various risk factors and pneumonia in young children, but no systematic reviews of published literature assessed the strength of association between the suspected risk factors and pneumonia. In this study, we aimed to assess the quality of available evidence and present summary estimates of the strength of association between the risk factors and severe pneumonia in children using meta-analysis.

## Methods

We systematically reviewed all literature published from January 1, 1990 through March 31, 2012 to identify studies with data on risk factors for pediatric pneumonia. We searched a variety of databases-Medline (Ovid), Embase, CINAHL and Global Health Library using combinations of key search terms: pneumonia, low birth weight, undernutrition, breast feeding, crowding, smoking, indoor air pollution, immunization, HIV etc. (full search terms are available in Supplementary material). Hand searching of online journals was also performed by examining the reference lists for relevant articles. We did not apply any language or publication restrictions. Relevant full-text articles in foreign language were translated to English using Google translator.

We defined an episode of severe pneumonia in hospital setting as any child hospitalized overnight with an admission diagnosis of pneumonia or bronchiolitis. In community-based studies, the presence of lower chest wall indrawing in a child with cough and difficulty breathing with increased respiratory rate for age was used to define a case, using the same cut off values as in the WHO's case definition (4,5). We recognized that the eligible studies used varying case definitions for the putative risk factors. We therefore grouped the risk factor definitions into categories and analyzed the association between risk factor and outcome for each of these categories (Table 1). We classified the risk factors into three groups based on the consistency and strength of association with severe ALRI:

**Table 1:** Case definitions for the risk factors for severe acute lower respiratory infections (ALRI) used in the retained studies

Risk factor	Definition
Low birth weight	Birth weight <2.5 kg irrespective of gestational age
Breastfeeding	1) Lack of exclusive breastfeeding 2) No breastfeeding 3) Less than 4 mo exclusive breastfeeding
Crowding	1)>7 people per household 2)>2 people sharing child’s bedroom 3)>2 people per room
Indoor air pollution	Use of biomass fuels for cooking or a description of indoor smoke
Undernutrition	1) Weight for age <2 standard deviations (SD) 2) Height for age <2 SD 3) Weight for height <2 SD
Incomplete immunization	Lack of immunization for measles in a child aged >11 mo
HIV	Confirmed presence of HIV in a child
Passive smoking	1) Smokers in house 2) Maternal smoking
Maternal education	No maternal education
Sex	Being male
Vitamin D deficiency	Presence of rickets
Preterm birth	Birth before 37 weeks gestation
Anemia	Mostly serum hemoglobin <11 g/dL
Zinc	Protection offered by zinc supplementation
Daycare center attendance	Attendance at daycare center
Birth interval	Birth interval <24 mo
Previous pregnancy	>3 previous pregnancies
Previous illness	Previous history of ALRI
Vitamin A deficiency	Serum retinol <0.7 µmol/L

(i) those that consistently (ie, across all identified studies) demonstrated an association with severe ALRI, with a significant meta-estimate of the odds ratio, would be classified as “definite”;

(ii) those demonstrating an association in the majority (ie, in more than 50%) of studies, with a meta-estimate of the odds ratio that was not significant, would be classified as “likely;” and

(iii) those that were sporadically (ie, occasionally) reported as being associated with severe ALRI in some contexts were classified as “possible.” This classification is consistent with the one originally used by Rudan et al (2).

**Figure 1:**
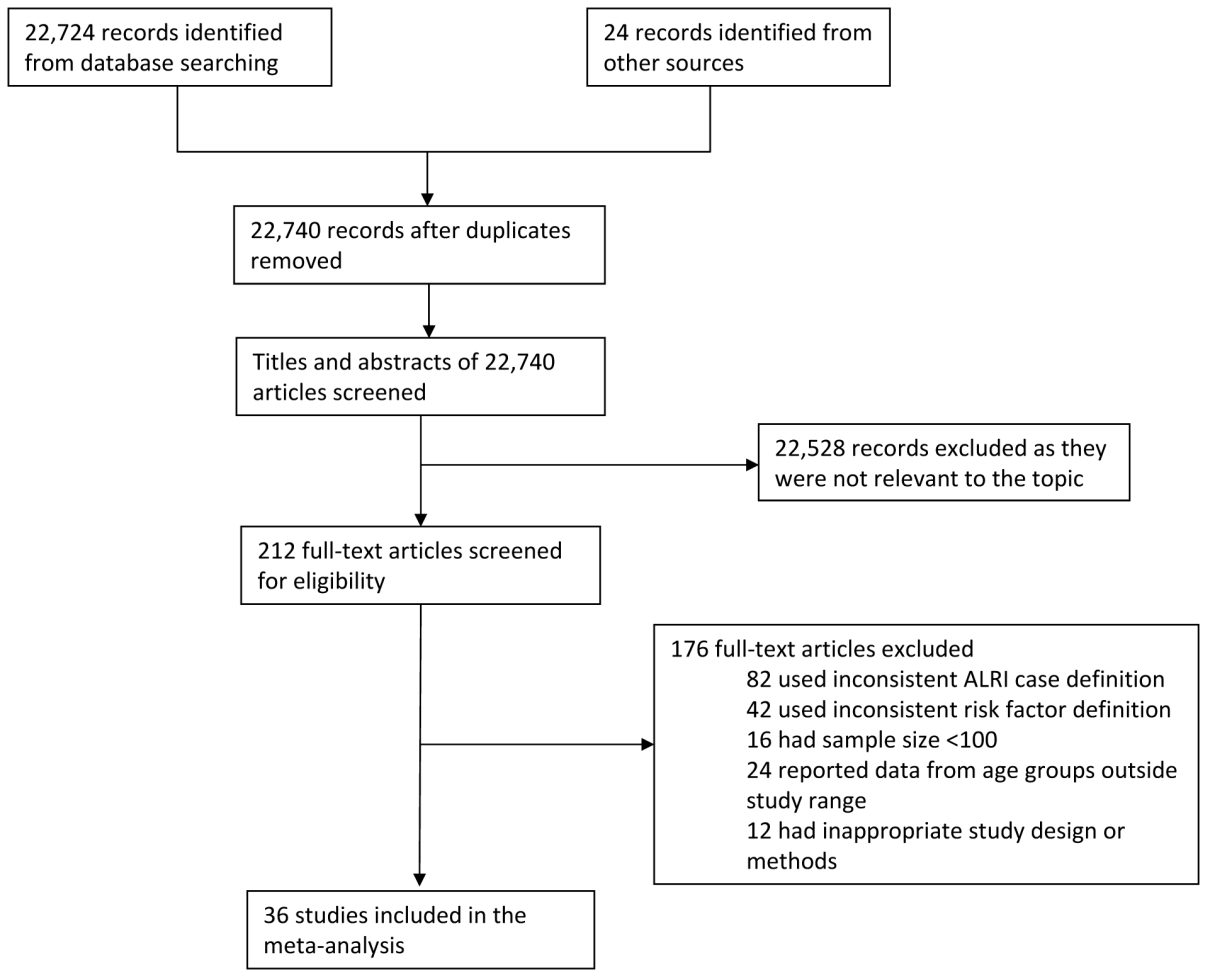
Flow diagram for selection of studies.

We included studies that reported severe pneumonia in children under five years of age (Table 2). Eligible study designs included randomized control trials (RCTs), observational studies (cohort, case-control, or cross-sectional) that assessed the relationship between severe pneumonia in children and any one of the putative risk factors. Studies were excluded if their sample size was less than 100 detected cases, if their case definitions did not meet our broad range of case definitions, or if the case definitions were not stated clearly and/or not consistently applied (Figure 1). Studies where health care workers went house to house to identify cases of pneumonia were considered as having active community-based case ascertainment. By contrast, studies where children with pneumonia presented to a health facility were considered as having passive hospital-based case ascertainment.

**Table 2:** Criteria for inclusion and exclusion of the reviewed studies

Inclusion criteria	Exclusion criteria
• Studies on children hospitalized (overnight) with an admission diagnosis pneumonia and bronchiolitis odds ratio presence of lower chest wall indrawing in a child with cough and difficulty breathing with increased respiratory rate for age	• Case definitions not clearly stated or inconsistently applied
• Risk factors defined as stated in Table 1	• Inappropriate control population – eg, children hospitalized for acute medical conditions (other respiratory conditions, diarrhea, meningitis, sepsis, etc.)
• Studies with sample size ≥100	• Study designs – surveys or case series
• Studies in children aged below five years	• Methods for statistical analysis not clearly reported
• Study designs – randomized control trials or observational studies (case-control or cohort)	
• Studies reporting results using univariate or multivariate analysis	

**Figure 2:**
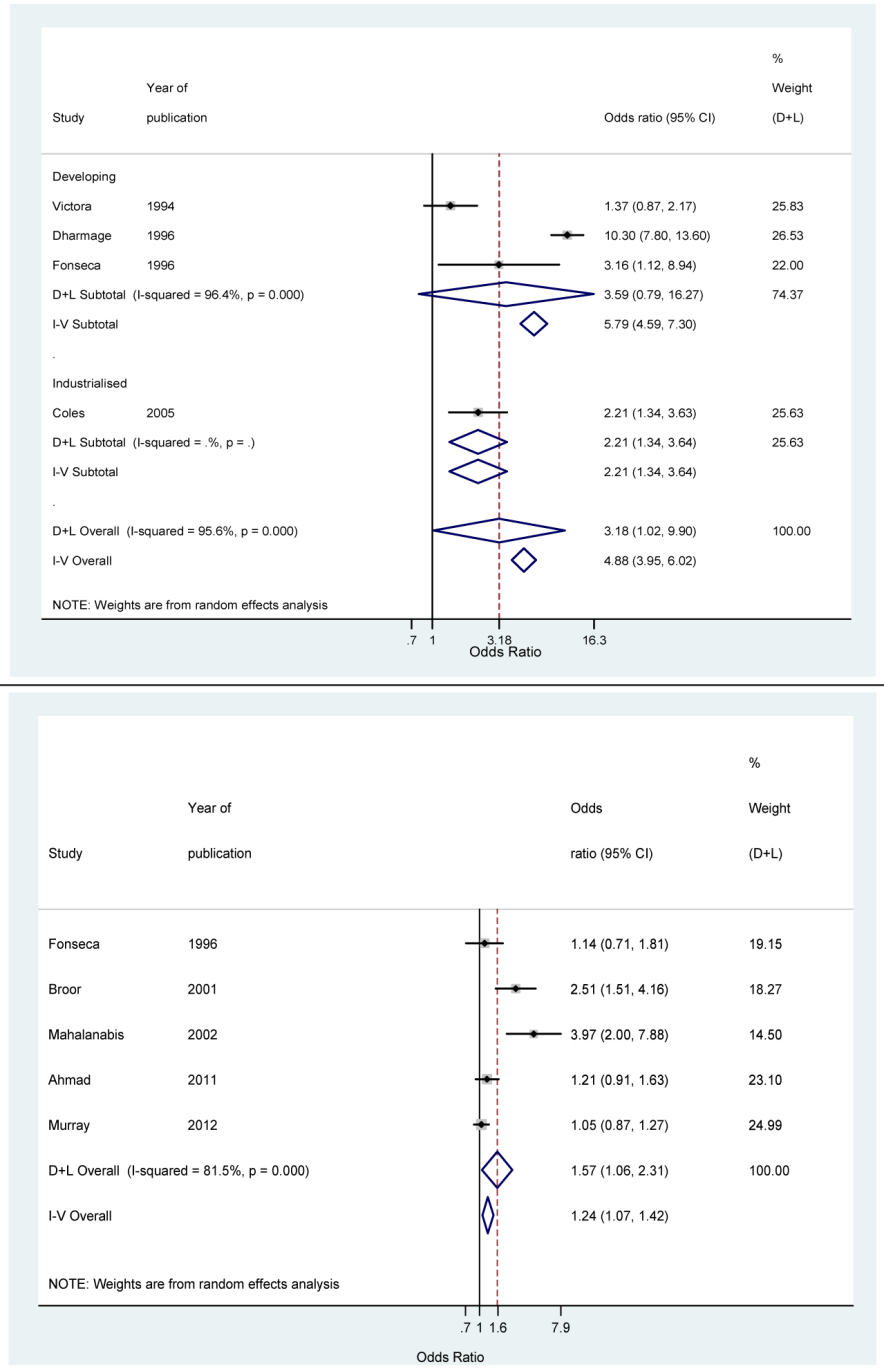
Forrest plots from meta-analysis odds ratio estimates for the following risk factors: low birth weight (top) and indoor air pollution (bottom). D+L indicates meta-estimates from random effects model; I-V from fixed effects model.

The included studies used either multivariate or univariate analyses to report the association between the putative risk factors and the outcome, ie severe pneumonia. Since the multivariate design takes into account the interaction with other risk factors and potential confounders, we decided to report the results of the meta-analysis of these data separately. We decided that if there was significant heterogeneity in the data, ie, I^2^>80%, (corresponding to *P* < 0.005) (6), then we would report the meta-estimates from the random effects model (7). Importantly, we hypothesized that the effects of the risk factors were likely to be different in developing countries and industrialized countries. Because of this, we decided to report the results separately for developing (Table 3) and industrialized countries (Table 4). We extracted all relevant information from each retained study (Supplementary Table S2(supplementary Table 2)) and assessed the quality of included studies using a modified GRADE scoring system (Supplementary Tables S1(supplementary Table 1)) (8). Briefly, we assessed each article against the GRADE criteria and calculated the overall score for each article. We then calculated the cumulative score for each risk factor after accounting for the included studies (Supplementary Table S3(supplementary Table 3)). We used Stata 11.2 (StataCorp, College Station, TX, USA) for the meta-analysis (Figure 2; Supplementary Figure S1(supplementary Figure 1)).

**Table 3:** Meta-estimates for risk factors for severe acute lower respiratory infections (ALRI) in children aged below 5 y in developing countries (meta-estimates for all risk factors except HIV are from multivariate analyses only)

Risk factor	No. of studies included	Meta-estimate (95% CI)	*P* value from meta analysis	I^2^ for heterogeneity (%)
Low birth weight	3	3.6 (0.8-16.3)	<0.005	96.4
Breastfeeding				
lack of exclusive breastfeeding	8	2.7 (1.7-4.4)	<0.005	87.5
no breastfeeding	4	2.8 (1.0-7.7)	<0.005	91.5
<4 mo breastfeeding	4	2.6 (1.6-4.4)	<0.005	79.0
Crowding				
crowding (>7 persons/household)	2	1.9 (1.5-2.5)	0.829	0
crowding (>2 persons sharing child’s bedroom)	4	2.2 (1.8-2.7)	0.475	0
indoor air pollution	5	1.6 (1.6-2.3)	<0.005	81.5
Undernutrition				
weight for age <2 standard deviation (SD)	6	4.5 (2.1-9.5)	<0.005	84.7
height for age <2 SD	2	4.8 (3.7-6.1)	0.819	0
weight for height <2 SD	4	2.8 (1.8-4.3)	0.051	61.4
incomplete immunization	5	1.8 (1.3-2.5)	0.021	65.4
HIV*	2	4.6 (2.2-9.7)	<0.005	90.2
Passive smoking				
smokers in house	4	2.5 (0.8-8.0)	<0.005	97.9
maternal smoking	3	2.7 (1.0-7.8)	<0.005	84.4
No maternal education	5	1.9 (1.0-3.7)	0.011	69.4
Sex (being male)	6	1.5 (1.0-2.3)	<0.005	89.9
Vitamin D deficiency (rickets)	3	7.3 (2.5-21.5)	0.002	83.6
Preterm births	2	1.3 (0.8-2.1)	0.582	0
Zinc deficiency	2	0.5 (0.3-0.9)	0.250	24.4
Day care attendance	2	8.0 (3.6-17.7)	0.188	42.2
Birth interval <24 mo	2	1.4 (0.9-2.2)	0.765	0
Birth order ≥3 (previous pregnancies >3)	2	3.0 (2.0-4.6)	0.788	0
History of ALRI	4	2.3 (1.8-3.1)	0.372	4.2

**Table 4:** Meta-estimates for risk factors for severe acute lower respiratory infections (ALRI) in children aged below 5 y in industrialized countries (meta-estimates are from multivariate analyses only)

Risk factor	No. of studies included	Meta-estimate (95% confidence interval)	*P* value from meta analysis	I^2^ for heterogeneity (%)
**Breastfeeding**				
Lack of exclusive breastfeeding	2	1.3 (0.2-8.4)	<0.005	88.9
No breast feeding	2	1.3 (0.2-8.4)	<0.005	88.9
**Crowding**				
Overcrowding (>2 people per room)	2	2.3 (1.3-4.0)	0.411	0
**Passive smoking**				
Smokers in house	2	2.1 (1.3-3.6)	0.733	0
**Undernutrition**				
Height for age <2 standard deviation (SD)	2	1.3 (0.5-3.4)	<0.005	91.3

Received: March 9, 2013

Accepted: April 10, 2013

## Results

Our initial literature search yielded 22 740 studies with suitable data (Figure 1). After screening of the titles and abstracts and a subsequent full text review against our eligibility criteria, we identified 36 studies with suitable data. Twenty seven studies reported odds ratios using multivariate analysis and 25 studies reported odds ratios using univariate analysis. Studies reporting the association between HIV and vitamin A deficiency and severe ALRI reported risk estimates using only univariate analysis. The quality of studies reporting the strength of association between risk factors (exposure to low birth weight, indoor air pollution, and lack of maternal education) and outcome (severe ALRI) was good (average cumulative modified GRADE score greater than or equal to 2). However, the quality of studies reporting the association between some other risk factors (incomplete immunization at one year and prematurity) was poor (average cumulative modified GRADE score less than 1) (Supplementary Table S3(supplementary Table 3)).

### Definite risk factors

The following risk factors were consistently associated with severe ALRI:

*Low birth weight*. Four hospital-based studies (9-12) (three from developing region) reported an association between low birth weight and severe ALRI using multivariate analysis. The overall and developing region risk-ratio meta-estimate were 3.2 (95% CI 1.0 to 9.9) and 3.6 (95% CI 0.8 to 16.2), respectively (Figure 2; Table 3). Three additional studies (13-15), one from developing region, reported odds ratios for low birth weight and severe ALRI using univariate analysis. When data from these studies was combined with the data from studies using multivariate analysis, the odds ratio meta-estimate was 3.2 (95% CI 1.0 to 10.0) and 1.8 (95% CI 1.3 to 2.7) for developing and industrialized regions, respectively.

*Indoor air pollution exposure*. Five studies (10,16-19), all from developing region, reported an association between exposure to indoor air pollution (use of solid and biomass fuels) and severe ALRI using a multivariate analysis. The overall odds ratio meta-estimate was 1.6 (95% CI 1.1 to 2.3) (Figure 2; Table 3). Three additional studies (13,20,21) reported the association using a univariate analysis. The inclusion of these studies did not alter the odds ratio meta-estimate.

**Figure 3:**
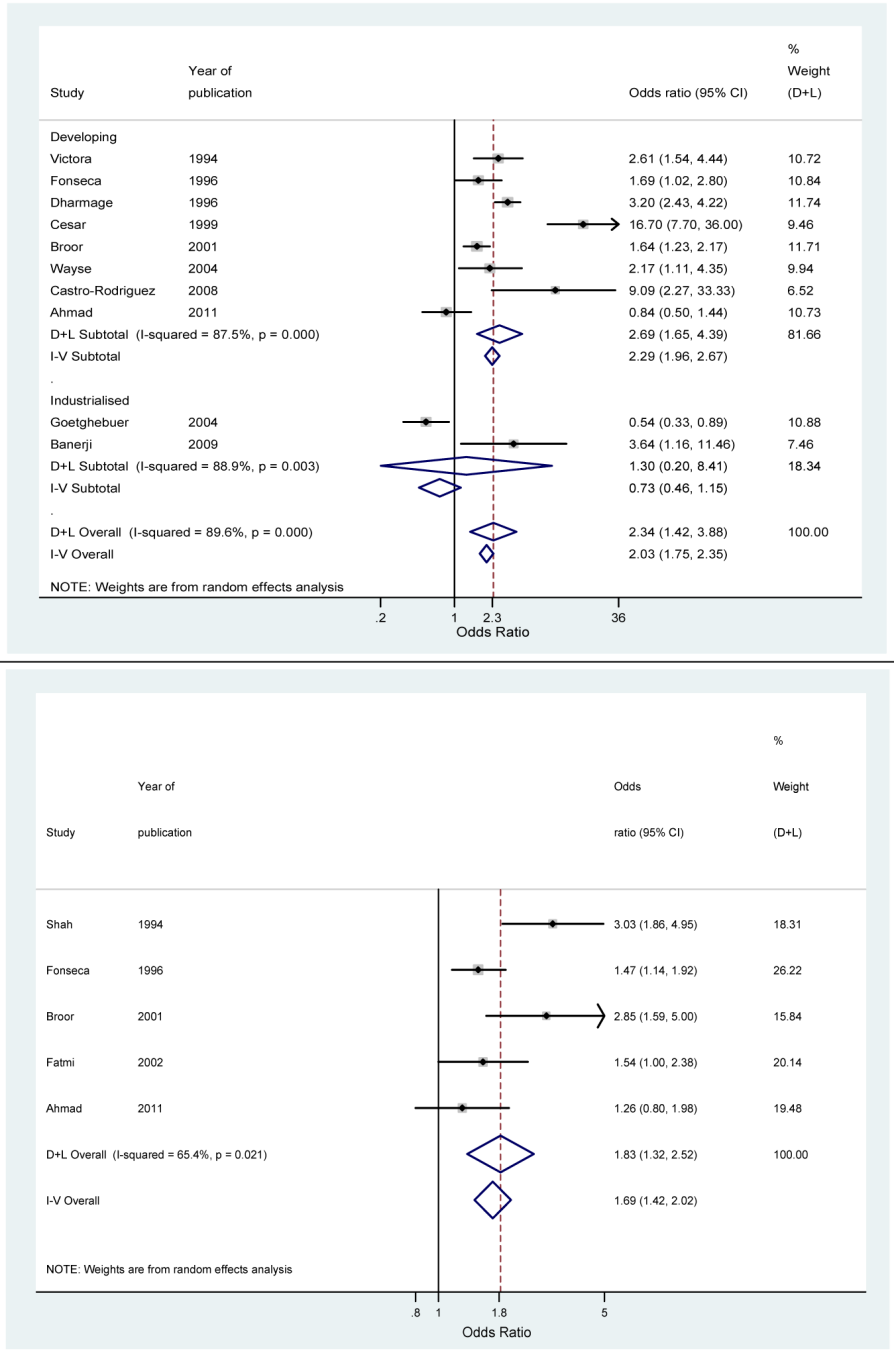
Forrest plots from meta-analysis odds ratio estimates for the following risk factors: lack of exclusive breastfeeding (top) and incomplete immunization (bottom). D+L indicates meta-estimates from random effects model; I-V from fixed effects model.

*Breastfeeding*. Ten hospital-based studies (9-11,14,16,19,22-25), 8 from developing countries, reported an association between lack of exclusive breastfeeding (defined as only breast milk in the first four months of life) and ALRI using a multivariate analysis. The odds ratio meta-estimate for developing and industrialized regions were 2.7 (95% CI 1.7 to 4.4) and 1.3 (95% CI 0.2 to 8.4), respectively (Figure 3; Table 3; Table 4). Six additional studies (12,13,15,26-28) (4 from developing countries) reported the association between lack of exclusive breastfeeding and severe ALRI using a univariate analysis. The addition of those studies did not have any substantial effect on the odds ratio meta-estimate. Six studies (9,10,14,19,22,25), 4 from developing regions, reported the association between no breastfeeding and severe ALRI. These studies showed an odds ratio meta-estimate similar to lack of exclusive breastfeeding. Four studies (11,16,23,24) from developing region reported an association between partial breastfeeding (less than four months of breast feeding) and severe ALRI. The odds ratio meta-estimate was again similar to that for lack of exclusive breastfeeding. Four studies (13,15,27,28), 3 from developing and 1 from industrialized countries, reported odds ratios using univariate analysis. Inclusion of those studies did not alter the odds ratios.

*Incomplete immunization.* We defined incomplete immunization as lack of immunization for measles at the end of the first year of life since bronchopneumonia is a common complication in children with measles, and has a high case fatality rate (29-31). Five studies (10,16,19,20,32), all from developing region, reported an association between incomplete immunization and severe ALRI. The overall odds ratio meta-estimate was 1.8 (95% CI 1.3 to 2.5). Three additional studies (13,27,33) from developing region also reported this association. Inclusion of these studies did not alter the odds ratio meta-estimate. Two studies from industrialized region (15,34) reported the association between incomplete immunization and severe ALRI using a univariate analysis. The odds ratio meta-estimate, however, did not report a significant association between the risk factor and the outcome. (Figure 3; Table 3; Table 4)

**Figure 4:**
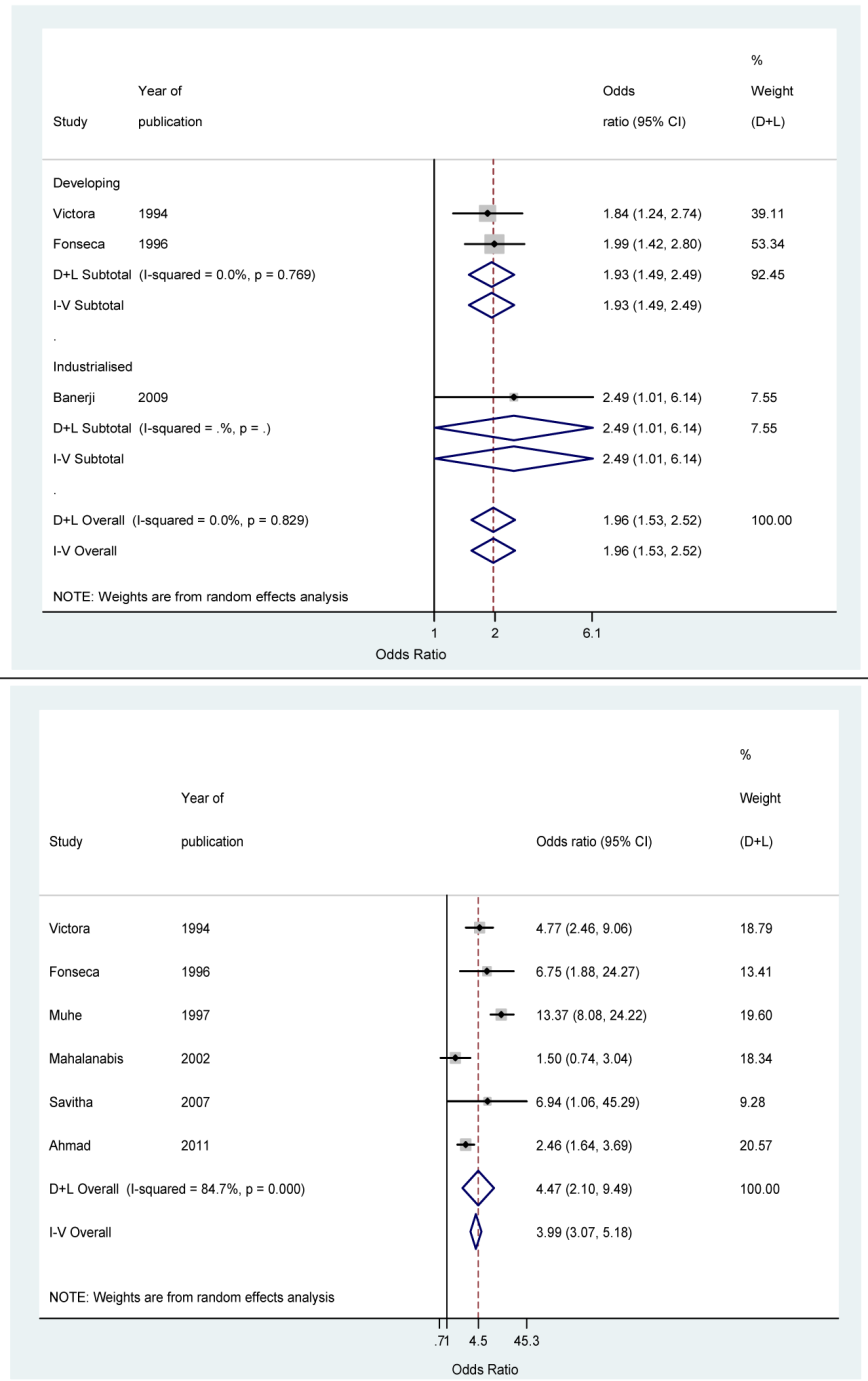
Forrest plots from meta-analysis odds ratio estimates for the following risk factors: crowding (top) and undernutrition (bottom). D+L indicates meta-estimates from random effects model; I-V from fixed effects model.

*Crowding*. Three studies (9,10,25) (two from developing countries) reported an association between crowding (>7 persons per household) and severe ALRI using a multivariate analysis. The odds ratio meta-estimate for the developing region was 1.9 (95% CI 1.5 to 2.5) (Figure 4; Table 3). Three additional studies (13,16,32) from developing region reported an association between crowding (>7 persons per household) and severe ALRI using a univariate analysis. Inclusion of these studies did not alter the odds ratio meta-estimate. Four studies (11,20,28,35), all from developing regions, defined crowding as more than 2 persons sharing a child’s bedroom. These studies, using a multivariate analysis, reported an odds ratio meta-estimate of 2.2 (95% CI 1.8 to 2.7) (Figure 4; Table 3). Two additional studies (9,14), one from developing region, reported the association using univariate analysis. Again, addition of this study did not alter the odds ratio meta-estimate.

Seven additional studies (9,11,13,15,17,27,34), 5 from developing region, reported an association between crowding (>2 persons per room) and severe ALRI using a multivariate or univariate analysis. The overall odds ratio meta-estimate for the developing region was similar to those using other case definitions, with odds ratio meta-estimate 2.0 (95% CI 1.2 to 3.2). The odds ratio meta-estimate for the two studies from industrialized region was similar, with odds ratio meta-estimate 2.3 (95% CI 1.3 to 4.0).

*Undernutrition*. Undernutrition has been variously defined in the literature: underweight (weight for age <2 standard deviations [SD]), stunted (height for age <2 SD), and wasted (weight for height <2 SD). Six studies (9,10,17,19,27,36), all from developing regions, reported an association between being underweight and risk for severe ALRI using a multivariate analysis. The overall odds ratio meta-estimate was 4.5 (95% CI 2.1 to 9.5). Four additional studies (12,15,23,32), 2 from developing region, reported this association using a univariate analysis. Inclusion of these studies decreased the overall odds ratio estimate by about 30%, with odds ratio meta-estimate 3.2 (95% CI 1.6 to 6.4). There was no significant association between being underweight and the risk of severe ALRI in industrialized countries, with odds ratio meta-estimate 1.2 (95% CI 0.6 to 2.5). Four studies (10-12,15), two from developing region, reported an association between stunting and severe ALRI using a multivariate analysis. Three additional studies (9,23,32), all from developing regions, reported this association using a univariate analysis. The overall odds ratio meta-estimate (for studies using univariate and multivariate analysis) for the developing regions was 2.6 (95% CI 1.5 to 4.6). In the case of the two studies from industrialized region using multivariate analysis, the overall odds ratio meta-estimate was similar to the risk for being underweight, with odds ratio meta-estimate 1.3 (95% CI 0.5 to 3.4).

Four studies (10,16,32,36), all from developing countries, reported an association between wasting and severe ALRI. The overall odds ratio meta-estimate from these four studies was 2.8 (95% CI 1.8 to 4.3). Six additional studies (9,11,12,15,33,37), 4 from developing region, reported this association using a univariate analysis. Inclusion of these studies did not substantially alter the overall odds ratio meta-estimate for developing countries. The two studies from developing countries again did not report a significant association. (Figure 4; Table 3).

*HIV infection*. Only two studies (38,39) reported an association between HIV and severe ALRI. Both studies were from sub-Saharan Africa and used univariate analysis. The overall odds ratio meta-estimate was 4.6 (95% CI 2.2 to 9.7). (Supplementary Figure S1(supplementary Figure 1)).

### Likely risk factors

The following risk factors where associated with severe ALRI in a majority of included studies:

*Passive smoking*. We observed an inconsistent association between the presence of smokers in the house and severe ALRI in the studies included in this review. While 4 studies (11,20,40,41) (three from developing region) reported an association between the presence of smokers in the house and severe ALRI using a multivariate analysis, 2 (15,34) failed to do so. Overall, the odds ratio meta-estimate (from studies using a multivariate analysis) did not report a significant association between the risk factor and the outcome, with odds ratio meta-estimate 2.4 (95% CI 1.0 to 5.8). The evidence of an association between the presence of smokers in the house and severe ALRI was again inconsistent when studies using a univariate analysis were considered. While only 2 studies (14,33) (one from developing region) demonstrated an association, 3 studies (9,13,32), all from developing region, did not do so. Inclusion of these univariate studies did not substantially alter the overall odds ratio meta-estimate. Three studies (25,42,43), all from the industrialized region, reported an association between maternal smoking and severe ALRI using a multivariate analysis. The overall odds ratio meta-estimate was 2.7 (95% CI 1.0 to 7.8). However, another study (14) from industrialized region, did not report such an association when the data were analyzed using a univariate analysis. Inclusion of this study did not substantially alter the overall odds ratio meta-estimate.

*Lack of maternal education* The association between the lack of maternal education and severe ALRI was inconsistent. While one study (28) from developing region reported an association using a multivariate analysis, 5 studies (10,12,17,19,22), 1 from developing region, did not do so. The overall odds ratio meta-estimate was 1.6 (95% CI 1.0 to 2.6). When studies using a univariate analysis were considered, only 3 of the 4 studies (18,27,32) (all from developing region) reported an association between the absence of maternal education and severe ALRI. Inclusion of these univariate studies did not substantially alter the overall odds ratio meta-estimate.

*Sex.* The association between male sex and severe ALRI was inconsistent. Only 3 of the 6 studies (10,11,20,22,28), all from developing region, reported an association between male sex and severe ALRI using a multivariate analysis. Overall, the odds ratio meta-estimate was 1.5 (95% CI 1.0 to 2.3). Only 3 of 7 studies (9,14,17,18,32,37), 6 from developing region, reported such an association using a univariate analysis. Inclusion of these univariate studies did not substantially alter the odds ratio meta-estimate.

*Vitamin D deficiency*. Vitamin D deficiency was defined broadly as the presence of clinical rickets in 4 of the 5 studies that investigated an association between the risk factor and severe ALRI (the one remaining study defined this as Vit D_3_<22.5 nmol/L). Three studies (19,23,36), all from developing regions, demonstrated an association between the presence of rickets and severe ALRI using a multivariate analysis. The odds ratio meta-estimate was 7.3 (95% CI 2.5 to 21.5). Two studies (27,34) (one from developing region) demonstrated a similar association using a univariate analysis. Inclusion of these studies did not substantially affect the odds ratio meta-estimate.

*Preterm birth*. Two studies (20,22), both from developing region, did not demonstrate an association between preterm birth and severe ALRI using a multivariate analysis. The overall odds ratio meta-estimate was not significant, with odds ratio 1.3 (95% CI 0.8 to 2.1). However, three studies (13,14,33) (2 from developing region) demonstrated such an association when using a univariate analysis. Inclusion of these studies significantly altered the odds ratio meta-estimate to 1.9 (95% CI 1.3 to 2.8).

*Anemia.* Anemia was inconsistently defined in the 5 studies. Only 2 studies defined this using the measurement of hemoglobin (Hb) in blood (but used different cut-off values of Hb to define anemia). Two studies (12,44), one from developing region, demonstrated an association between anemia and severe ALRI using a multivariate analysis. The overall odds ratio meta-estimate was 3.9 (95% CI 2.4 to 6.3). Two of the 3 studies (23,27,37) (all from developing countries), also demonstrated an association between anemia and severe ALRI using a univariate analysis. Inclusion of these univariate studies did not significantly alter the odds ratio meta-estimate.

*Zinc deficiency*. Two studies (45,46), both from developing region, reported an inverse association between zinc supplementation and severe ALRI using a multivariate analysis, with odds ratio meta-estimate 0.5 (95% CI 0.3 to 0.9). However, one study (47) from developing region did not report a protective effect of zinc supplementation on severe ALRI using a univariate analysis. Inclusion of this study did not substantially alter the odds ratio meta-estimate.

### Possible risk factors

The following risk factors were sporadically associated with severe ALRI in the identified studies:

*Day care*. Two studies (9,10) from developing region reported an association between daycare attendance and severe ALRI using a multivariate analysis. The overall odds ratio meta-estimate was 8.0 (95% CI 3.6 to 17.7). However, another study (15) from industrialized region did not report such an association when using a univariate analysis. Inclusion of this study significantly altered the odds ratio meta-estimate to 3.7 (95% CI 0.7 to 20.2).

*Birth interval*. Two studies (10,22), both from developing regions, did not report an association between birth interval (<24 months) and severe ALRI using a multivariate analysis, with odds ratio meta-estimate 1.4 (95% CI 0.9 to 2.2). Two more studies (9,11) from developing region also failed to observe such an association when using a univariate analysis. Inclusion of these studies did not significantly alter the odds ratio meta-estimate.

*Birth order.* Two studies (10,22), both from developing region, reported an association between three or more previous pregnancies and severe ALRI using a multivariate analysis. The overall odds ratio meta-estimate was 3.0 (95% CI 2.0 to 4.6). However, another study (9) (again from developing regions) using a univariate analysis did not report such an association. Addition of this study did not substantially alter the odds ratio meta-estimate, which amounted to 2.1 (95% CI 1.2 to 4.4).

*Previous history of ALRI.* Four of the 5 studies (9,10,15,28,32) included in this review reported an association between the risk factor (history of ALRI) and outcome (severe ALRI) using a multivariate analysis. The overall odds ratio meta-estimate did not demonstrate a significant association, with odds ratio meta-estimate 1.7 (95% CI 0.8 to 3.4).

*Vitamin A deficiency.* Only one study, from industrialized region (12), reported an association between vitamin A deficiency (serum retinol <20 µg/dL) and severe ALRI. However this association was not significant, with OR 1.01 (95% CI 0.47 to 2.16).

## Discussion

Our study is the first comprehensive attempt to systematically assess the effect of a multitude of possible risk factors on severe ALRI in children aged less than five years. We identified, in total, 19 risk factors, which had been reported to be associated with severe ALRI in the published literature. We observed a consistent significant association between 7 risk factors (low-birth-weight, undernutrition, indoor air pollution, incomplete immunization at one year, HIV, breastfeeding, and crowding) and severe ALRI (definite risk factors). We also observed that 7 risk factors (parental smoking, lack of maternal education, vitamin D deficiency, male sex, preterm births, anemia, and zinc deficiency) had an inconsistent association with severe ALRI that was not significant (likely risk factors). We further observed that 5 risk factors (daycare, birth interval, birth order, previous history of ALRI, and vitamin A deficiency) were sporadically reported to be associated with severe ALRI (possible risk factors).

There was considerable variation in the quality of eligible studies included in this review (Supplementary Table S3(supplementary Table 3)). We used a modified GRADE scoring (8) to assess the quality of included studies. The modified grade score varied from 0.9 (in the case of preterm births) to 4 in the case of daycare attendance. However, we recognize that this score only partially reflects the quality of the included studies. The included studies used variable case definitions. While we grouped together studies using similar case definitions, in some cases there was a substantial variability. For example, in the case of low birthweight, while all studies used a case definition of birthweight less than 2500 g, Victora et al (9) used a cut-off of <2000 g to define low-birth-weight. Similarly, there was a considerable difference in the age groups of included participants across studies. While 19 studies reported outcomes for 0-59 months age range, 17 studies only included a narrow age range (eg, 0-11 months or 0-23 months). Since we pooled the data from different age ranges (but using the same case definitions), we may have over-estimated the odds ratio meta-estimates if a substantial number of the included studies were for a lower age range.

Although we only included the studies with a sample size ≥100, there was considerable variability in the sample size of the included studies- ranging from 146 (26) to 350 648 (43). This is reflected in the wide confidence intervals for some of the reported odds ratio estimates included in the meta-analysis. The included studies were significantly heterogeneous.

Twenty eight of the 36 included studies obtained data on the presence of risk factors only from interviewing mothers of the eligible subjects. Eight studies (26,34,36-39,43,44) used either records/laboratory diagnosis alone or in combination with maternal history for data collection. This introduced several biases in the studies, such as recall bias, interviewer bias, and misclassification bias. We also observed that there was considerable variation in the adjustment for potential confounders in the included studies. While 2 studies did not adjust for potential confounders (20,33), 9 studies did (15,16,19,25,37,39,40,42,43). The majority of the identified risk factors appeared to be related to poverty and hence significant collinearity is likely to exist among them. Only sixteen (9-11,13,15,17,18,22,28,35,39,40,42,43,46,47) of the 36 included studies adjusted for poverty as a confounder.

Hospitalization for acute lower respiratory infections in young children poses a substantial burden on health services, especially in developing countries. In spite of this, the evidence regarding the risk factors for this major burden of disease is in many cases sparse, of variable quality, and not generalizable. However, since the majority of these risk factors are potentially preventable, governments should consider what action can be taken to decrease the prevalence of these risk factors. This, along with increasing coverage of vaccines for *Streptococcus pneumoniae and Haemophilus influenzae* type B, should substantially decrease the burden of childhood pneumonia in developing countries. Moreover, the odds ratio meta-estimates reported in this review should be useful for modeling the global, regional, and national estimates of severe ALRI if the national/regional prevalence of these risk factors are known (2). Future studies should investigate the role of poverty, HIV, and other risk factors currently classified “likely” or “possible” risk factors for ALRI, and should attempt to obtain more precise estimates of risk for “definite” risk factors by studying larger samples in diverse settings and by more careful measurement and analysis of possible confounding factors.
