# Supplementary material for: Risk factors for severe acute lower respiratory infections in children – a systematic review and meta-analysis
Source: Croat Med J. 2013 Apr;54(2):110–21. doi: 10.3325/cmj.2013.54.110 (PMC3641871; doi:10.3325/cmj.2013.54.110)
Supplement: Supplementary Table 2 [file CroatMedJ_54_s003.pdf]

Supplementary table 2: Studies investigating risk factors for severe pneumonia in children under five years of age. (LBW=Low Birth Weight; BF=Breastfeeding; PI=Previous ALRI; CR=Crowding; PS=Passive Smoking; IAP=Indoor Air Pollution; DC=Day Care; MN=Malnutrition; IM=Immunization; ME=Maternal Education; AN=Anemia; VDd=Vitamin D deficiency; GE=Gender; VAd=Vitamin A deficiency; Zd=Zinc deficiency; PB=Preterm Birth; BO=Previous Pregnancy; BI=Birth Interval)

Location; site characteristics; period of study (reference)	Case ascertainment	Study design	Controls	Age group	Sample size	Risk factors	Type of analysis
Buenos Aires, Argentina; urban; 1984-7 (13)	Passive hospital	Case-control	154 ambulatory patients attending immunization and well-baby clinics. Gender, age, nutritional status, socioeconomic level, season and place of residence matched	0-59 months	1032	LBW, CR, PS, IAP, IM, PB, BF	Univariate
Basse, Gambia; rural (41)	Active community	Cohort		0-59 months	587	PS	Multivariate
Naples (Campania), Italy; urban; 1983-88 (26)	Passive hospital	Case-control	73 age matched infants admitted to same ward. Matched for age and month of admission. Controls had the following diagnoses: anemia (5), cardiac disease (7), inguinal hernia and hydrocele (25), congenital clubfoot (12), meningitis (3), otitis (7), febrile seizures (4) and urinary tract infection (10).	0-5 months	146	BF	Univariate
Trivandrum (Kerala), India; urban; 1991 (20)	Passive hospital	Case-control	100 out-patients with ARI, with no symptoms of severe pneumonia and not requiring admission for these symptoms at 5 days follow up. No effort made to match controls.	0-59 months	400	CR, PS, IAP, IM, GE, PB	Multivariate and Univariate
Porto Alegre;Rio Grande do Sul, Brazil; urban; 1990 (9)	Passive hospital	Case-control	500 age-matched children from same neighborhood	0-23 months	1020	DC, BF, PI, CR, PS, MN, ME, GE, BO, BI, LBW	Multivariate and Univariate
Oslo, Norway; urban; 1992 (42)	Passive hospital	Cohort		0-11 months	3238	PS	Multivariate
Colombo, Sri Lanka; urban (11)	Passive hospital	Case-control	100 age-matched children on surgical wards	0-59 months		LBW, BF, CR, PS, MN, GE, BI	Multivariate and Univariate
Fortaleza (Ceara State), Brazil; urban; 1989-90 (10)	Passive hospital	Case-control	650 age-matched children from same neighborhood	0-23 months	1300	DC, BF, PI, CR, IAP, MN, IM, ME, GE, BO, BI, LBW	Multivariate
Addis Ababa, Ethiopia; urban; 1989-93 (36)	Passive hospital	Case-control	500, age-matched and admitted to hospital within 3 months of case	0-59 months	1000	MN, VDd	Multivariate

Location; site characteristics; period of study (reference)	Case ascertainment	Study design	Controls	Age group	Sample size	Risk factors	Type of analysis
Pelotas, Brazil; urban; 1993 (22)	Passive hospital	Case-control	2391 community based children. Cases and controls were ratified for age but not matched at individual level	0-11 months	1141	BF, ME, GE, PB, BO, BI	Multivariate and Univariate
Stockholm (Gothenburg and Malmo), Sweden; urban; 1990-4 (43)	Health records	Cohort		0-11 months	350648	PS	Multivariate
Ballabgarh (Haryana), India; rural; 2003-5 (16)	Passive hospital	Case-control	311 healthy children below 5 years of age attending immunization clinic	0-59 months	512	BF, CR, IAP, MN, IM	Multivariate and Univariate
Basrah, Iraq; urban; 1996-7 (33)	Passive hospital	Case-control	250 children attending outpatient department with non-severe ARI that did not require admission for symptoms on follow-up	2-59 months	298	PS, MN, IM, PB	Univariate
Calcutta (West Bengal), India; urban; 1997-8 (17)	Passive hospital	Case-control	135 children attending hospital for immunization. Stratum matched for age.	2-35 months	262	CR, IAP, MN, ME, GE	Multivariate and Univariate
Dakshinpuri (New Delhi), India; urban (47)	Active community	Randomized control trial	1241 children received placebo; comparable for age, anthropometry, child feeding practices, morbidity in previous 24 hours, socioeconomic characteristics and plasma zinc concentration	6-30 months	2482	ZI	Univariate
Gilgit (Gilgit District), Pakistan; urban; 2000 (32)	Passive hospital	Case-control	187 children with ‘coughs and colds’, with no signs of pneumonia development at 3 days	0-59 months	446	PI, CR, PS, MN, IM, ME, GE	Multivariate and Univariate
Dhaka, Bagladesh; urban; 1997 (45)	Active community	Randomized control trial	149 children received placebo	1-6 months	301	ZI	Multivariate
Matlab Upzila (Chandpur), Bangladesh; rural (46)	Active community	Cohort		5-12 months	799	ZI	Multivariate
Oxford (Oxfordshire), England; urban; 1995-9 (14)	Passive hospital	Case-control	872 randomly selected children with same date of birth and sex, with no history of admission for ALRI selected from hospital database	0-11 months	496	LBW, BF, CR, PS, GE, PB	Univariate
Indapur (Maharashtra), India; urban; 2002 (23)	Passive hospital	Case-control	70 health children younger than 5 living in same area as cases, attending for vaccination. Not matched with cases	0-59 months	150	BF, MN, AN, VDd	Multivariate and Univariate
Sao Paulo, Brazil; urban (35)	Passive hospital	Case-control	347 children, crudely matched for age, selected from same neighborhood.	2-59 months	732	CR	Multivariate

Location; site characteristics; period of study (reference)	Case ascertainment	Study design	Controls	Age group	Sample size	Risk factors	Type of analysis
Beersheba (Negev), Israel; rural; 2001-2 (12)	Passive hospital	Case-control	529 healthy Bedouin children less than 5 seen for regular check up who had no diagnosis of pneumonia in previous 28 days. Matched for age and month of enrolment.	0-59 months	825	LBW, BF, MN, ME, AN, Vad, BF	Multivariate and Univariate
Soweto (Johannesburg), South Africa; 1998-2001 (38)	Passive hospital	Randomized control trial	19,914 children received placebo	0-72 months	39,836	HIV	Univariate
Pelotas, Brazil; urban; 1997-8 (28)	Passive hospital	Case-control	152 age matched community based controls with no history of ALRI	0-11 months	777	BF, PI, CR, ME, GE	Multivariate and Univariate
Mysore (Kamataka), India; urban; 2005 (27)	Passive hospital	Case-control	104 healthy children, aged 1-59 months, who were siblings of admitted children for non respiratory complaints during study period	0-59 months	208	BF, CR, MN, IM, ME, AN, VDd	Multivariate and Univariate
Santiago, Chile; urban (24)	Passive hospital	Cohort		0-11 months	188	BF	Multivariate and Univariate
Manhica (Maputo), Mozambique; rural; 2004-6 (37)	Passive hospital	Cohort		0-23 months	4838	MN, AN, GE	Univariate
Baffin Island (Nunavut), Canada; rural; 2002-3 (25)	Passive hospital	Case-control	101 community based, age matched children with no previous history of ALRI. Recruited during study period	0-23 months	220	BF, CR, PS	Multivariate
Nha Trang and Ninh Hoa (Khanh Hoa), Vietnam; mixed; 2006 (40)	Active community	Cross-sectional study	24,145 children with no history of pneumonia in the past 1 year	0-59 months	24781	PS	Multivariate and Univariate
Pokhara (Kaski District), Nepal; urban; 2006-7 (44)	Passive hospital	Case-control	140 age and sex matched children with no LRT symptoms	1-60 months	280	AN	Multivariate
ZVITAMBO Project; Harare; Zimbabwe; 1997-2000 (39)	Passive hospital	Cohort	9207 not exposed infants, 2661 exposed but not infected infants	0-35 months	14,110	HIV	Univariate
San Marcos, Guatemala; rural; 2002-4 (21)	Active community	Randomized control trial	253 control children	0-17 months	534	IAP	Univariate
Auckland, New Zealand; urban; 2002-4 (15)	Passive hospital	Case-	351 community controls, with similar ages and ethnicity, but not individually	0-59 months	428	DC, LBW, BF, PI, CR, PS,	Multivariate and

Location; site characteristics; period of study (reference)	Case ascertainment	Study design	Controls	Age group	Sample size	Risk factors	Type of analysis
		control	matched			MN, IM, GE	Univariate
Peshawar (Khyber-Pakhtunkhwa), Pakistan; urban; 2007-8 (19)	Passive Hospital	Case-control	200 children of similar age, sex and socioeconomic status hospitalized for causes other than respiratory infections, and also from immunization clinics etc.	2-60 months	300	BF, IAP, IM, ME, GE, VDd	Multivariate
Saskatoon (Saskatchewan), Canada; urban; 2007-8 (34)	Passive hospital	Case-control	92 unmatched controls of same age without respiratory infection	0-59 months	197	CR, PS, IM, VDd	Multivariate and Univariate
Kamalapur (Dhaka), Bangladesh; urban; 2004-5 (18)	Active community	Cohort		0-59 months	6079	IAP, ME, GE	Multivariate and Univariate
