# Supplementary material for: Risk factors for severe acute lower respiratory infections in children – a systematic review and meta-analysis
Source: Croat Med J. 2013 Apr;54(2):110–21. doi: 10.3325/cmj.2013.54.110 (PMC3641871; doi:10.3325/cmj.2013.54.110)
Supplement: Supplementary Table 1 [file CroatMedJ_54_s004.pdf]

Supplementary table S1: Modified GRADE scoring

**Criteria**		**Score**
Design	Randomised control trial	+2 points
	Cohort study	+1 point
	Case control study	+0.5 points
	Cross sectional study	0 points
Quality of control group	Good	+1 point
	Average	-1 point
	Poor	-2 points
Sample size	>500 subjects	+0.5 points
	300-500 subjects	0 points
	<300 subjects	-0.5 points
Odds Ratio	<0.2	+2 points
	0.2-0.49	+1 point
	0.5-2	0 points
	2-4.9	+1 point
	>5	+2 points
Confounding factors	All accounted for	+1 point
	Some accounted for	0 points
	Not accounted for	-1 point
Geographical spread of studies	Good geographical distribution	0 points
	Studies in one region only	-0.5 points
