# Supplementary material for: Risk factors for severe acute lower respiratory infections in children – a systematic review and meta-analysis
Source: Croat Med J. 2013 Apr;54(2):110–21. doi: 10.3325/cmj.2013.54.110 (PMC3641871; doi:10.3325/cmj.2013.54.110)
Supplement: Supplementary Table 3 [file CroatMedJ_54_s005.pdf]

Supplementary table 3: Quality assessment of included studies reporting risk factors for severe ALRI according to GRADE system.

Risk factor	Number of studies	Case ascertainment	Limitations	Consistency	Modified GRADE score*	Generalisability to population of interest
Low birth weight	7	All passive, hospital based	Only some studies use radiology to confirm diagnosis. Potential selection and recall bias.	Consistent. All studies show association between LBW and ALRI.	2.75	Good. Studies performed in 6 different countries in both developed and developing world.
Breastfeeding	16	All passive, hospital based	Potential selection and recall bias. Possible reverse causality bias.	Consistent. All studies show association between lack of breastfeeding and ALRI.	1.75	Good. Studies performed in 11 different countries in both developed and developing world.
Crowding	15	All passive, hospital based	Definitions variable. Potential selection and response bias.	Association consistently demonstrated.	1.2	Good. Studies performed in 8 different countries in developed and developing world.
Passive smoking	14	12 passive hospital	Definitions variable. Potential for selection and recall bias. Response bias.	Inconsistent. Association not always demonstrated.	1.56	Good. Studies performed in 13 countries in developed and developing world.
Indoor air pollution	7	6 passive hospital	Definitions variable. Potential for selection bias.	Association not demonstrable in all studies.	2	Fairly good-representative of most developing country locations. 3 studies each from India (different locations) and South America (different countries).
Malnutrition	12	12 passive hospital	Possibility of reverse causality, recall bias and selection bias.	Consistent. Majority of studies demonstrate association.	1.55	Good. Studies performed in 8 countries in both developed and developing world.

Immunization	11	All passive, hospital based	Possible reverse causality, response bias and selection bias. Immunizations studies variable (e.g 'partial immunization' vs measles vs DPT)	All studies of univariate analysis demonstrated effect.	0.9	Good. Studies performed in 8 different countries in both developed and developing world.
Maternal education	10	9 passive hospital	Definitions variable. Selection and response bias.	Association not always demonstrated. All studies performed in developing countries.	2.21	Fairly good- representative of most developing country locations. 4 studies performed in Brazil and 5 in different locations in Asia.
Day care attendance	3	3 passive hospital	Selection bias.	Consistent. All show significance at univariate and multivariate level.	4	Poor. Two performed in Brazil. One in New Zealand.
Birth interval	4	All passive hospital in developing countries in an urban setting	Selection bias and recall bias.	Consistent. Majority of studies show no association between birth interval and ALRI at either univariate or multivariate level.	2	Poor. 3 of the studies performed in Brazil. One performed in Sri Lanka.
Previous ALRI	5	All urban, passive hospital	Recall bias.	Consistent. Significant at both univariate and multivariate analyses.	1.8	Average. 3 of the studies performed in Brazil. Studies in both developed and developing world.
Anaemia	5	All passive, hospital based	Possibility of reverse causality. Definitions variable.	Consistent. All show significance at univariate and multivariate level.	3	Fairly good. Performed in 4 different countries.
Gender		12 passive hospital	Selection bias.	Inconsistent. Association not always demonstrated.		Good. Studies performed in 8 different countries in both developed and developing world.

	13				1.33	
Vitamin D deficiency	5	All urban, passive hospital	Possibility of reverse causality.	Consistent. All show significance at univariate and multivariate level.	1.17	Fairly good. Studies performed in 4 different countries, all developing.
Preterm birth	6	All passive, hospital based	Selection bias and recall bias.	Inconsistent. Association not always demonstrated.	0.25	Good. Performed in 5 different countries in both developed and developing world.
Previous pregnancies	3	All passive hospital in an urban setting.	Definitions vary. Selection bias.	Inconsistent. Association not always demonstrated.	3	Poor. All studies performed in Brazil.
Zinc	3	All active community	Possibility of reverse causality and selection bias.	Consistent. Two studies show zinc supplementation to be protective at multivariate level.	2.75	Poor. 2 studies carried out in Bangladesh, one in India.
Retinol	1	Passive hospital	40% of cases lost to follow up. Possibility of reverse causality.	Identified as a risk factor for ALRI.	2	Poor. Only study available performed in Israel.
HIV	2	1 passive hospital and 1 active community	Multiple confounders. Selection bias.	Consistently identifies association between HIV and ALRI.		Poor. Both studies performed in Southern Africa.

*Multivariate studies only
