# Supplementary material for: Risk factors for severe acute lower respiratory infections in children – a systematic review and meta-analysis
Source: Croat Med J. 2013 Apr;54(2):110–21. doi: 10.3325/cmj.2013.54.110 (PMC3641871; doi:10.3325/cmj.2013.54.110)

Supplementary figure S1: Meta-analysis of the effect of HIV/AIDS on the occurrence of severe ALRI in children under five years of age.

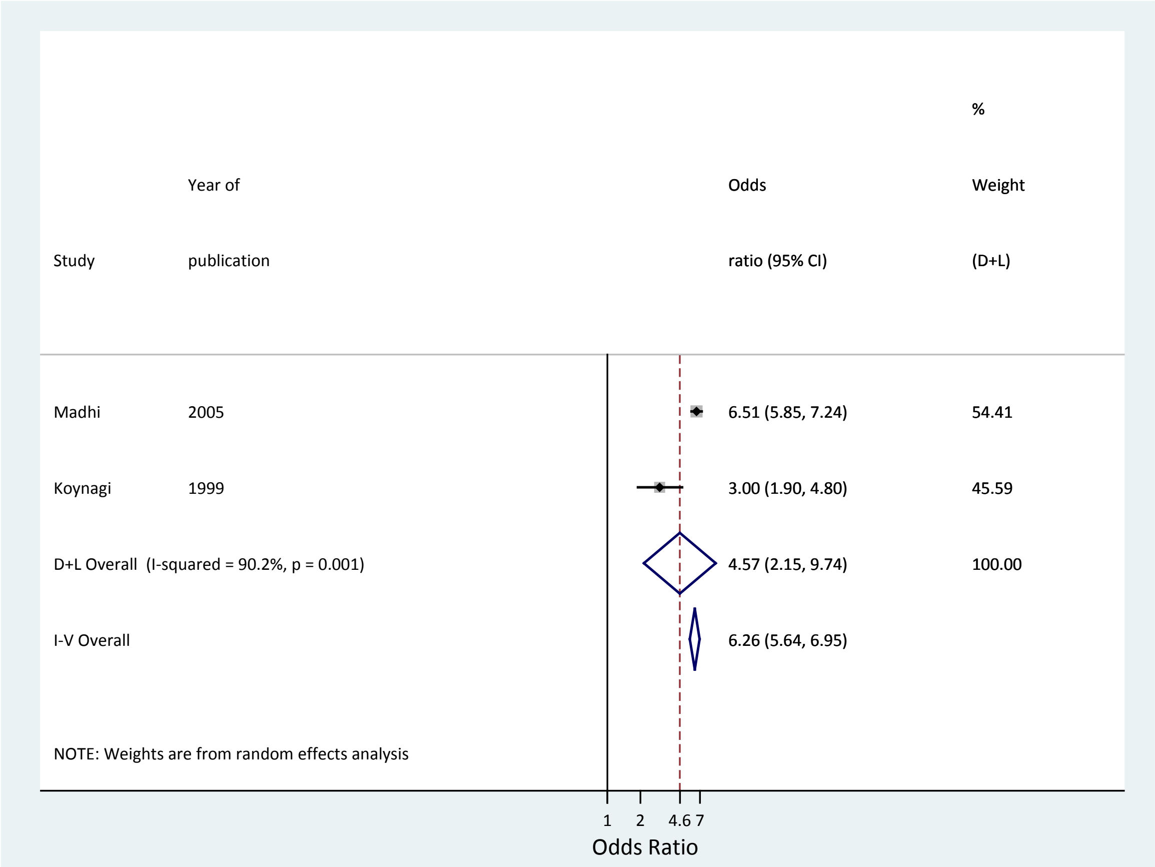

Supplement: Supplementary Figure 1 [file CroatMedJ_54_s006.pdf]
